# Capturing the Surface Texture and Shape of Pollen: A Comparison of Microscopy Techniques

**DOI:** 10.1371/journal.pone.0039129

**Published:** 2012-06-12

**Authors:** Mayandi Sivaguru, Luke Mander, Glenn Fried, Surangi W. Punyasena

**Affiliations:** 1 Institute for Genomic Biology, University of Illinois, Urbana, Illinois, United States of America; 2 Department of Plant Biology, University of Illinois, Urbana, Illinois, United States of America; Emory University, United States of America

## Abstract

Research on the comparative morphology of pollen grains depends crucially on the application of appropriate microscopy techniques. Information on the performance of microscopy techniques can be used to inform that choice. We compared the ability of several microscopy techniques to provide information on the shape and surface texture of three pollen types with differing morphologies. These techniques are: widefield, apotome, confocal and two-photon microscopy (reflected light techniques), and brightfield and differential interference contrast microscopy (DIC) (transmitted light techniques). We also provide a first view of pollen using super-resolution microscopy. The three pollen types used to contrast the performance of each technique are: *Croton hirtus* (Euphorbiaceae), *Mabea occidentalis* (Euphorbiaceae) and *Agropyron repens* (Poaceae). No single microscopy technique provided an adequate picture of both the shape and surface texture of any of the three pollen types investigated here. The wavelength of incident light, photon-collection ability of the optical technique, signal-to-noise ratio, and the thickness and light absorption characteristics of the exine profoundly affect the recovery of morphological information by a given optical microscopy technique. Reflected light techniques, particularly confocal and two-photon microscopy, best capture pollen shape but provide limited information on very fine surface texture. In contrast, transmitted light techniques, particularly differential interference contrast microscopy, can resolve very fine surface texture but provide limited information on shape. Texture comprising sculptural elements that are spaced near the diffraction limit of light (∼250 nm; NDL) presents an acute challenge to optical microscopy. Super-resolution structured illumination microscopy provides data on the NDL texture of *A. repens* that is more comparable to textural data from scanning electron microscopy than any other optical microscopy technique investigated here. Maximizing the recovery of morphological information from pollen grains should lead to more robust classifications, and an increase in the taxonomic precision with which ancient vegetation can be reconstructed.

## Introduction

Pollen grains are an indispensable record of Earth's vegetation and have multiple applications. They provide unique information on aspects of plant evolution such as the origin and radiation of the angiosperms (flowering plants) [1]–[4], and offer glimpses of plant life during periods in Earth history characterized by major environmental change and extinction [5]–[7]. Pollen grains are used to examine the influence of climate on the Earth's vegetation [8], [9], and data on the response of different plant species to past climatic changes are used to inform conservation efforts [10]. Pollen is also a notorious allergen, and periods when the pollen of notably allergenic plants such as grass or ragweed is particularly abundant in the atmosphere can be identified by the analysis of air-borne pollen [11]. It is also serves as an important tool in forensic science and can help link people or objects to crime scenes [12].

The study of pollen is part of the discipline of palynology (the study of living and fossil spores, pollen and other palynomorphs), and in each of these examples of palynological investigation, assemblages of pollen grains are generally split into groups on the basis of their morphology. These groups often correspond to the taxonomic grouping of the parent plants and may represent species, genera or families. In order to accurately define these morphological groups it is vital that the morphological information captured from individual specimens is as detailed and robust as possible. This is essential in order to reconstruct ancient vegetation with a high degree of taxonomic precision [13], [14]. The pollen of angiosperms ranges from ∼4.5 µm (the forget-me-not *Myosotis sylvatica*) to ∼200 µm (the pumpkin *Cucurbita pepo*) [15], and as a result of this small size the acquisition of morphological information from pollen grains is intimately linked to microscopy.

However, pollen grains present a significant challenge to the microscopist. The pollen exine, the outer layer of a pollen grain that is resistant to strong acids and bases and is primarily composed of the biopolymer sporopollenin [16], is strongly autofluorescent [17]. As a result, sporopollenin cannot be labeled using fluorophores or nano quantum dots as is standard in cell biology [18]. Further, the exine surface of pollen grains is often sculptured with elements that are smaller than the diffraction limit of light. The diffraction limit restricts the resolution of most high numerical aperture objectives to ∼250 nm in most practical situations [19], [20].

Routine palynological studies often involve the examination of thousands of individual pollen grains, and investigations of this type generally employ brightfield-transmitted light [17], [21]. In order to examine fine morphological differences between the pollen grains of different plant species, some workers have used contrast-enhancing techniques such as differential interference contrast microscopy (DIC) [22] and phase contrast microscopy [23], [24]. Fluorescence microscopy is emerging as a powerful tool in palynology. Widefield fluorescence has been used to identify reworked or poorly preserved specimens in samples of fossilized pollen grains [17] and apotome microscopy has been employed to investigate the shape of modern and fossil spruce pollen [25]. Confocal laser scanning microscopy has proved to be effective at revealing details of the ultrastructure of pollen walls in the absence of transmission electron microscopy [26], as well as providing information on the shape of pollen grains [27].

The purpose of this paper is to compare the performance of different optical microscopy techniques to provide information on the shape and surface texture of pollen grains. These are two crucial aspects of pollen morphology that have functional and taxonomic significance. The microscopy techniques that were compared are: widefield fluorescence, apotome [28], confocal and two-photon microscopy (reflected light techniques), and brightfield and DIC microscopy (transmitted light techniques). We also provide data from scanning electron microscopy (SEM) and a technique new to pollen microscopy: super-resolution structured illumination microscopy (SR-SIM). The SR-SIM technique has a two-fold improvement in resolution over conventional optical microscopy techniques [29] and is the only super-resolution optical microscopy technique that can image autofluorescent samples without labeling. Three pollen types with exine thickness ranging from thick to thin, and surface texture ranging from coarse to very fine, have been used to contrast the performance of each technique: *Croton hirtus* (Euphorbiaceae), *Mabea occidentalis* (Euphorbiaceae) and *Agropyron repens* (Poaceae).

## Results

### The performance of reflected light techniques with fluorescent beads

**Figure 1:**
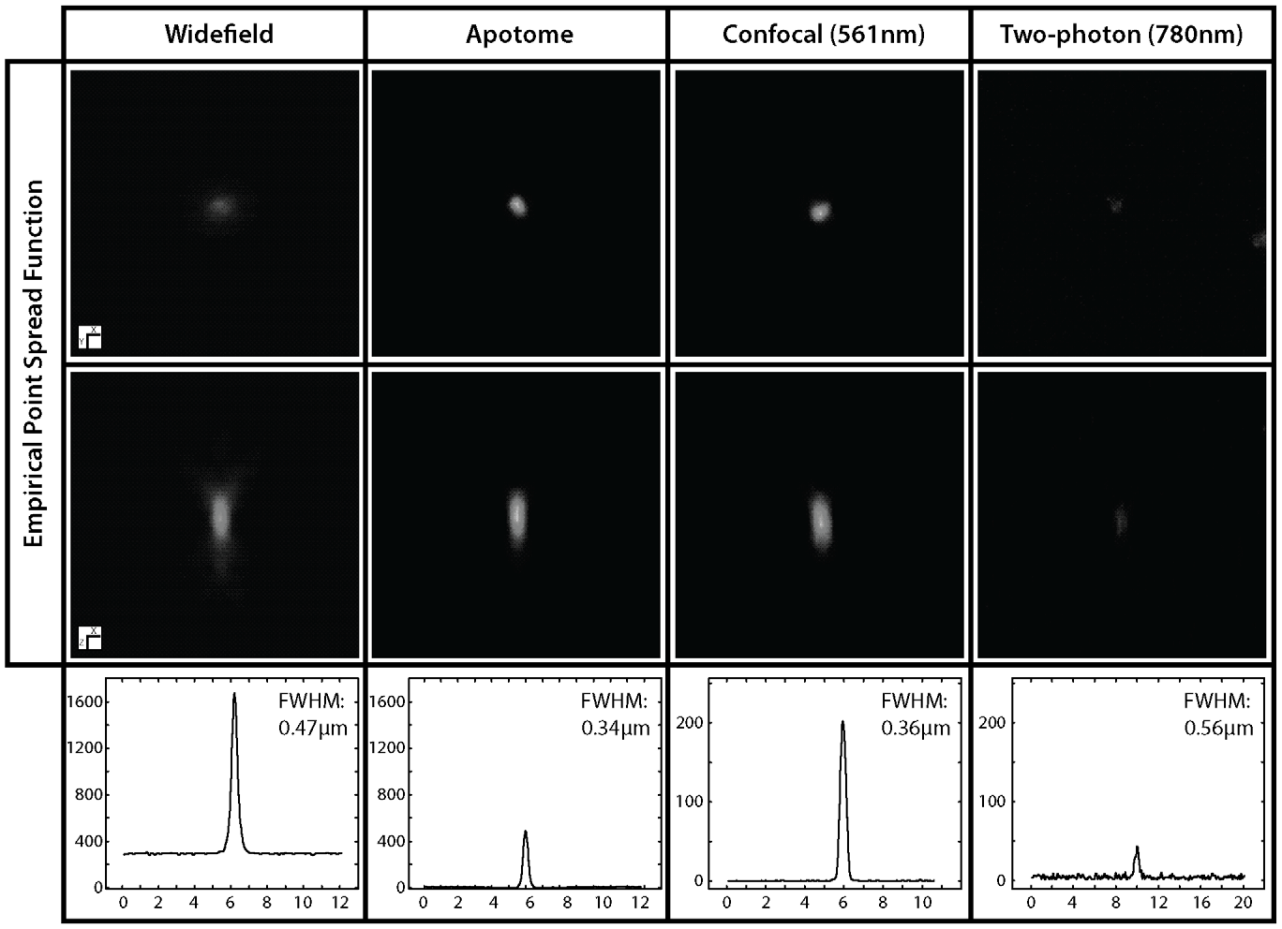
Point spread functions and resolution. Empirical point spread functions of ∼0.175 µm beads using widefield, apotome, confocal and two-photon microscopy techniques for a pair (two objectives each one for widefield-apotome pair and confocal-two photon pair) of Zeiss 63× plan apochromat 1.4NA lenses. Widefield and apotome images were acquired with a rhodamine filter (546/10 excitation filter). Images show a single bead in X-Y dimensions (upper tier) and X-Z dimensions (lower tier). Graphs show line profiles of pixel intensities across the centre of each bead in X-Y dimensions. FWHM = full width at half maximum. The point spread function at FWHM represents a measure of the resolution of a microscope [30], [32].

The full width of a PSF measured at half its maximum (FWHM) is a useful measure of the resolution of a microscope system [30]. This measure indicates that the microscopy techniques investigated here vary widely in lateral resolution (Figure 1). Two-photon microscopy (780 nm excitation) has the largest FWHM and hence the lowest resolution (560 nm) (Figure 1). Widefield microscopy has a FWHM of 470 nm, while apotome and confocal microscopy have very similar FWHMs (340 nm and 360 nm, respectively) (Figure 1).

### The shape of pollen grains

**Figure 2:**
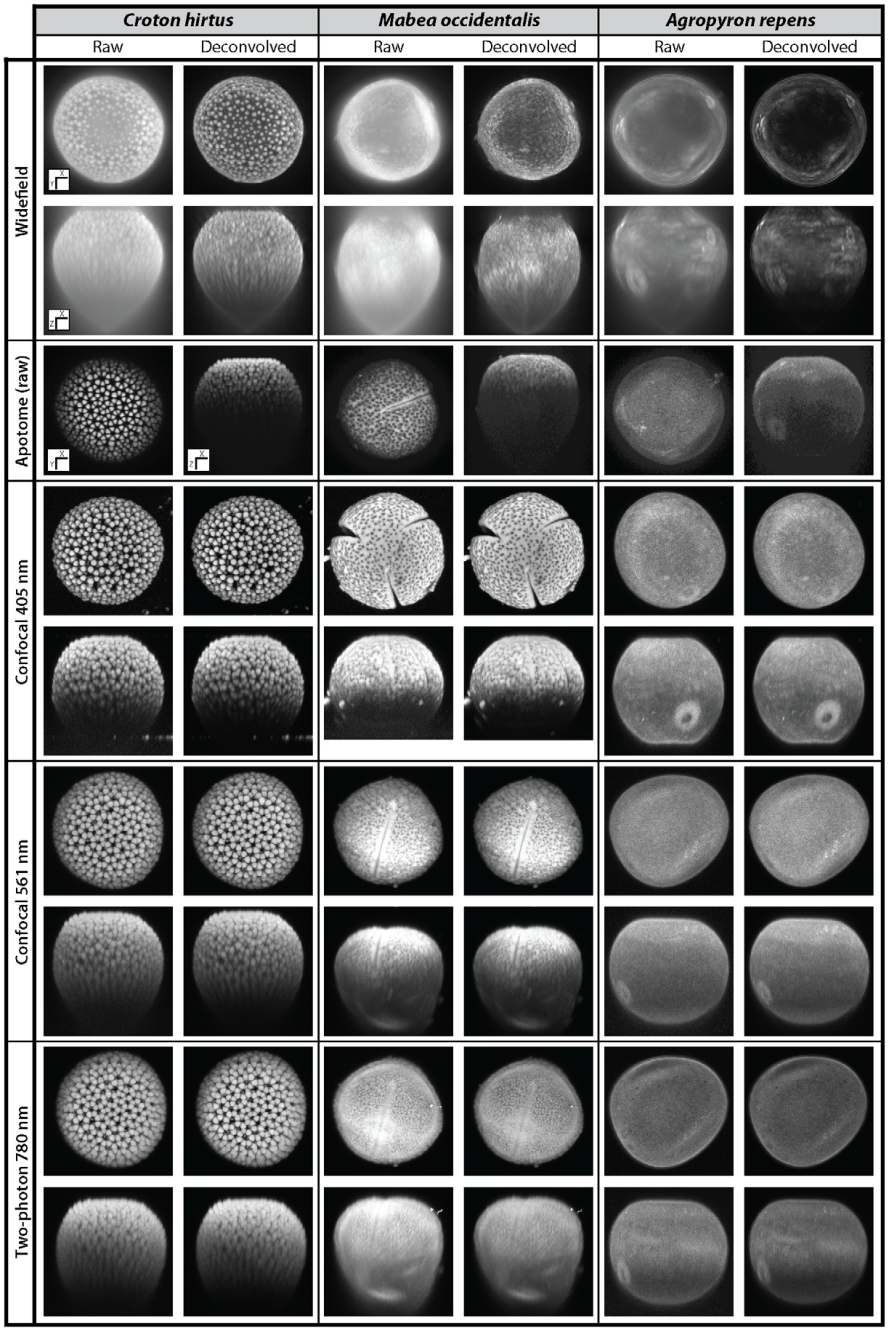
The shape of pollen grains. Maximum intensity projections of *Croton hirtus*, *Mabea occidentalis* and *Agropyron repens* viewed using widefield, apotome, confocal (405 nm and 561 nm) and two-photon fluorescence microscopy. Raw data are shown next to images produced following blind deconvolution. Note that only raw data are shown for apotome microscopy. Images displayed in the X-Y plane show the surface texture of each pollen type; images displayed in the X-Z plane show how the microscopy techniques differ in their ability to capture information on the shape of each pollen type. For each pollen type, images from widefield and apotome microscopy show the same pollen grain. Images from confocal 561 nm and two photon 780 nm microscopy show the same pollen grain. A unique pollen grain was used for confocal 405 nm. An orientation key is provided in the lower left-hand corner of the raw widefield data of *C. hirtus*, which applies to all other specimens shown (note the orientation of pollen grains imaged using apotome microscopy). The approximate size (diameter) of each pollen grain shown is as follows: *C. hirtus* ∼50 µm; *M. occidentalis* ∼50 µm; *A. repens* ∼40 µm.

None of the reflected light techniques investigated here (Table 1) provide a complete picture of the shape of all three pollen types (Figure 2, Table 2). The shape of *C. hirtus* is not entirely resolved by all of the techniques investigated here (Figure 2, compare Videos S1, S2, S3, S4, S5, S6, S7). In contrast, the shape of *A. repens* is well resolved by confocal (405 nm and 561 nm) and two-photon microscopy (Figure 2). *A. repens* is significantly fainter when imaged using apotome microscopy than when using confocal (405 nm and 561 nm) and two-photon microscopy (Figure 2). Widefield microscopy provides warped reconstructions, and this is highlighted by the shape of the deepest plane of each pollen grain, which tapers to a pronounced point (Figure 2). Such distortion is also evident in the apotome image of *M. occidentalis* (Figure 2). Deconvolved images contain less out-of-focus light than raw data and consequently have much sharper boundaries (due to improved signal-to-noise ratio) than images from raw data (Figure 2). This effect is most noticeable in the widefield images of all three pollen types, and the restoration of the surface texture of *C. hirtus* is particularly striking (Figure 2). However, although the warped voxels of *A. repens* were partially recovered, deconvolution did not substantially improve the recovery of pollen shape, especially among confocal techniques (Figure 2).

**Table 1:** General imaging parameters.

Techniques	Systems	Objective	Dimensions/(X, Y, Z) (µm)	Emission wavelengths	Detectors	Deconvolution	Image Analysis and Rendering
Widefield Fluorescence, Apotome (VH Grid) and DIC	Axiovert M200, Carl Zeiss, Jena, Germany	63× Planapochromat (1.4 NA)	1388×1024 (0.1, 0.1, 0.3)	ExD546/10; EmD610/60	Axiocam MRm 1388×1040	WF Blind-10 Constrained Iterations	Axiovision Interactive Measurement and Imaris Surpass-Volume Modules
Confocal Single photon (405 nm =and 561 nm) and Two photon (780 nm)	LSM 710, Carl Zeiss, Jena, Germany	63× Planapochromat (1.4 NA)	512×512 (0.1, 0.1, 0.3)	Ex405; Em 408–501 Ex561; Em 570–673 Ex780 (2-Photon); Em 570–670	Hamamatsu Multialkali PMTs	Confocal and Multiphoton Modality Blind-10 Constrained Iterations	Axiovision Interactive Measurement and Imaris Surpass-Volume Modules

Image acquisition parameters and hardware configurations for the optical microscopy techniques used in the study. All imaging parameters including digital zoom, laser power and gain were kept constant within a pollen class.

**Table 2:** Performance comparison of all tested microscopy techniques.

Microscopy Technique	Shape			Surface texture			Deep plane texture		
	*C. hirtus*	*M. occidentalis*	*A. repens*	*C. hirtus*	*M. occidentalis*	*A. repens*	*C. hirtus*	*M. occidentalis*	*A. repens*
RL_WF+BDC-Rh.filter	−−	−−−	−−−	++	++	+++	−−−	−−	+
RL_Apotome-Rh.filter	−−−	−−−	−−	+++	++	−	−−−	−−−	−−−
RL_Confocal+BDC-405 nm	−−−	−−−	++	−	−	++	−−−	−−−	−
RL_Confocal+BDC-561 nm	−	−−	++	+++	++	−−	−−−	+	−−
RL_Two photon+BDC-780 nm	−	++	+++	+	+	−−−	++	+	−−
TL_WF-DIC-Halogen	−−−	−−−	−−−	++	+	+++	−−−	−−−	+++
TL_Confocal+DIC-405 nm	−−−	−−−	−−−	−	−	+++	−−−	−−−	++
TL_Confocal-DIC-561 nm	−−−	−−−	−−−	++	++	++	+	++	++
TL_Two photon-DIC-780 nm	−−−	−−−	−−−	+	+	+	+++	++	+
TL_Brightfield-Halogen	ND	ND	ND	+	++	−	++	+	−

Qualitative table highlighting the relative ability of each microscopy technique to provide information on the shape and surface texture of *Croton hirtus* (coarse pattern), *Mabea occidentalis* (medium pattern) and *Agropyron repens* (near diffraction limited pattern). Abbreviations as follows: WF = Widefield; BDC = Blind Deconvolution; ND = Not Determined; Rh = Rhodamine; RL = Reflected Light; TL = Transmitted Light; +++ = Clear image of pollen shape/texture; −−− = Unclear image of pollen shape/texture.

### The texture of pollen grains

**Figure 3:**
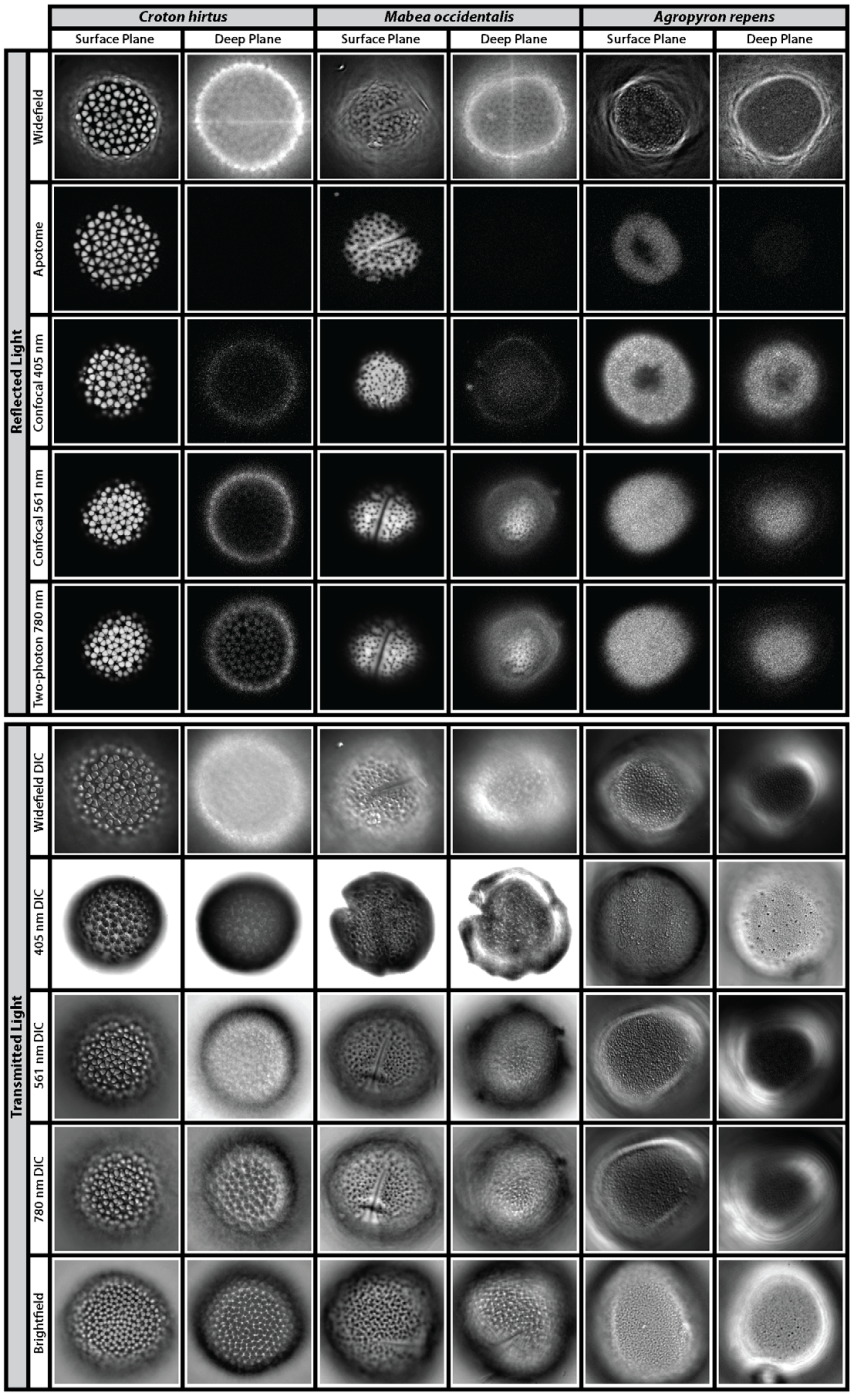
The surface texture of pollen grains. Optical sections of the pollen grains *Croton hirtus*, *Mabea occidentalis* and *Agropyron repens* viewed using reflected light (widefield, apotome, confocal and two-photon fluorescence microscopy) and transmitted light (widefield DIC, 405 nm and 561 nm DIC, 780 nm DIC and brightfield). Images from all reflected light techniques were deconvolved except apotome microscopy, which is represented by raw data. Images of surface planes are shown next to deep planes to highlight the depth of penetration of each technique. In order to acquire an image of *C. hirtus and M. occidentalis* using 405 nm DIC, the master gain was increased during acquisition. For each pollen type, images from widefield and apotome microscopy show the same pollen grain. Images from confocal 561 nm and two photon 780 nm microscopy show the same pollen grain. A unique pollen grain was used for confocal 405 nm. Images are displayed in min/max intensity profile. Pollen sizes as for Figure 2.

Most transmitted light techniques provide information on the texture of both surface planes and deep planes of each pollen types investigated here (Figure 3, Videos S8, S9, S10, S11, S12, S13, S14, S15). In contrast, reflected light techniques generally provide textural information from the surface planes of the three pollen types, but most of them fail to provide textural information from deep planes (Figure 3). For example, the surface plane texture of *C. hirtus* is well resolved by all reflected light techniques, but the deep plane texture of this pollen grain is only well resolved by two-photon microscopy (Figure 3, Video S6). The failure of apotome microscopy to provide textural information from the deep plane of any of the three pollen types is notable (Figure 3 and Video S5).

In reflected light the texture of *A. repens*, which comprises sculptural elements spaced near the diffraction limit of light (NDL), can be detected using widefield, apotome, and 405 nm confocal techniques. Widefield microscopy combined with deconvolution provides clear information on the surface plane texture of this pollen grain, as shown by discrete clusters of pixels with high intensity (Figure 3, Video S1). Apotome microscopy also provides some textural information from the surface plane of *A. repens* (Figure 2). Confocal microscopy with 405 nm excitation light provides reasonable textural information from the surface plane and possibly the deep plane of *A. repens* (Figure 3, Video S2) but confocal microscopy with 561 nm excitation light does not appear to have recovered textural information either from the surface plane or the deep plane of *A. repens* (Figure 3). Two-photon microscopy, which uses 780 nm excitation light, also fails to provide textural information either from the surface plane or the deep plane of *A. repens* (Figure 3).

There are subtle differences in the representation of the NDL texture of *A. repens* by the transmitted light techniques investigated here. In Widefield DIC and 405 nm DIC this texture is granulate [16], and consists mostly of discrete sub-circular clusters of high-intensity pixels that are dotted fairly evenly across the surface of the pollen grain (Figure 3, Videos S9, S10). However, under brightfield, 561 nm DIC and 780 nm DIC this texture is finely rugulate [16], and consists of more linear features spread across the pollen grain surface (Figure 3, Videos S12 and S14). It should be noted that while all transmitted light techniques provided reasonable information on both surface and deep plane textures of *M. occidentalis*, only two-photon DIC (780 nm) and brightfield microscopy provided textural information from the deepest plane of *C. hirtus* (Figure 3, Table 2 and compare Videos S11, S13 and S15). Phase contrast microscopy failed to provide significant textural information on any of the three pollen types investigated here (Figure S1). Hence, DIC was the only contrast-enhancing technique that was investigated.

**Figure 4:**
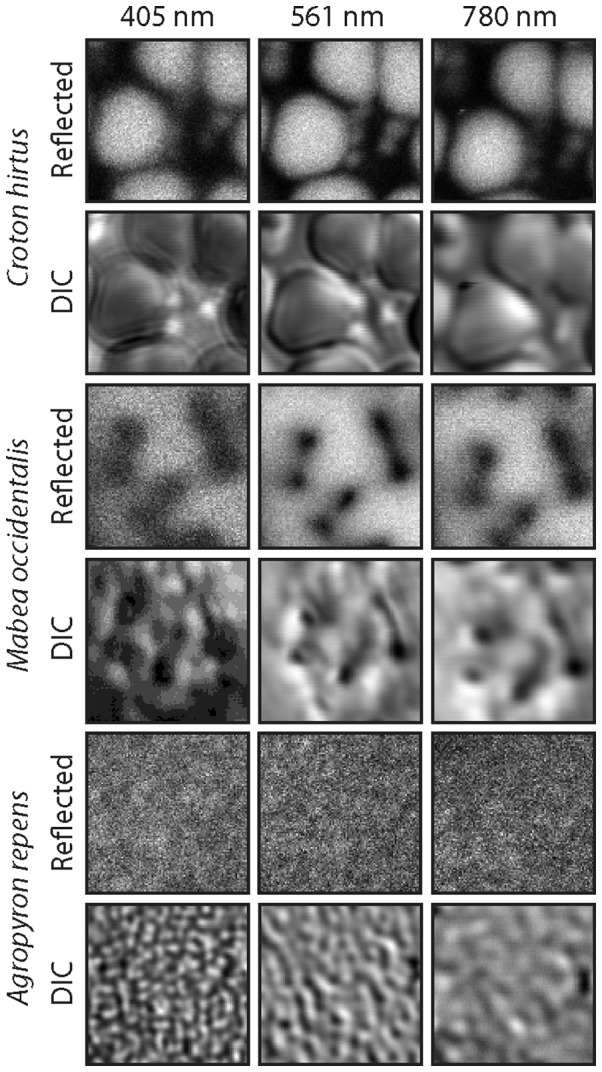
Cropped and zoomed-in views of the surface texture of *Croton hirtus*, *Mabea occidentalis* and *Agropyron repens*. Pollen grains imaged using confocal microscopy (405 nm and 561 nm), two-photon microscopy (780 nm) and DIC microscopy (405 nm, 561 nm and 780 nm). Images highlight the effect of wavelength, resolution, absorption and detectablility on the recovery of textural information from pollen grains. In order to acquire an image of *C. hirtus and M. occidentalis* using 405 nm DIC, the master gain was increased during acquisition. All images are raw data, measure 5 µm in the X-Y direction and are displayed in linear intensity profiles.

In order to summarize how the representation of surface texture changes between reflected and transmitted light, and with the wavelength of incident light, we have provided detailed cropped and zoomed views of the surface texture of each pollen grain (Figure 4). This figure shows raw data of a single pollen grain per pollen type. This data was acquired by simultaneously imaging three wavelengths of light (405, 561 and 780 nm) on the same pollen at the same time. Note that the PMT gain was more than doubled to reveal pollen grains using 405 nm DIC. This clearly shows how differences in the wavelength of incident light affect absorption and the signal-to-noise ratio among the three pollen types investigated here, especially with reference to *C. hirtus and M. occidentalis*. Images of these two pollen types produced by both reflected and transmitted light techniques have a low signal-to-noise ratio using the shorter wavelength of light (405 nm) (Figure 4, Video S3). In contrast, the confocal 561 nm, which is absorbed less by the pollen exine, produced outstanding images for the same two pollen species (Figure 3, Video S4). Despite the low signal evident in *C. hirtus and M. occidentalis*, the 405 nm DIC provided a higher resolution image of the NDL texture of *A. repens* than the 561 nm or 780 nm DIC (Videos S2, S10, S12 and S14). The change from granulate texture to fine rugulate texture in *A. repens* as the wavelength of light increases using DIC microscopy is clearly revealed (Figure 4).

### The impact of deconvolution

**Figure 5:**
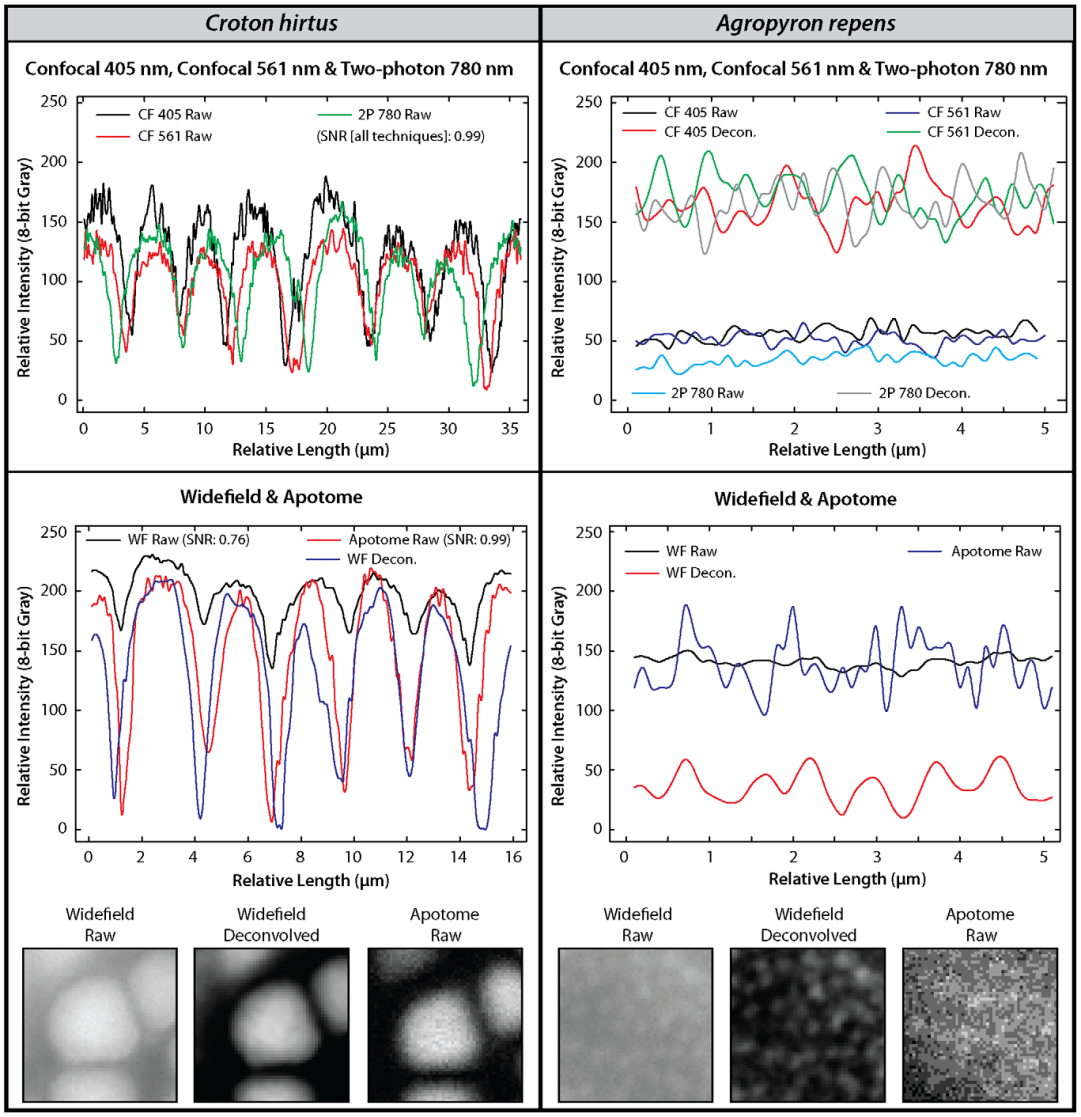
Effect of deconvolution. Cropped and zoomed-in images and intensity profiles on the surface texture of *Croton hirtus* and *Agropyron repens*, showing differences in the signal-to-noise ratio of each technique and the impact of blind deconvolution. CF = confocal; 2P = two-photon; WF = apotome; SNR = signal-to-noise ratio; Decon.  = deconvolved. SNR calculated as follows: x−y/x+y where x = the average of six maximum pixel intensity values from the peaks of the line profile, and y = the average of six minimum pixel intensity values from the troughs of the line profile (both areas randomly selected). In the case of *C. hirtus*, line profiles were traced on a single full Croton structure on the same pollen grain. In the case of *A. repens*, line profiles were traced on randomly selected regions of *A. repens* specimens. Data from widefield and apotome microscopy is from a single pollen grain. Data from confocal (405 nm and 561 nm) and two-photon microscopy is from a single pollen grain, but not the same specimen analysed used widefield and apotome microscopy. Intensity profiles were not drawn on precisely the same pixels in X-Y positions owing to the movement of pollen grains in the mounting media during the movement of slides between microscopes. Detailed images measure 5 µm in the X-Y direction and are displayed in linear intensity profiles.

In order to examine the effect of deconvolution on the recovery of textural information from pollen grains using reflected light microscopy techniques, we have taken line profiles of pixel intensity values from detailed images of *C. hirtus* and *A. repens* (Figure 5). In the case of *C. hirtus* we have also measured the signal-to-noise ratio of each reflected light technique on raw data (Figure 5). These analyses show that for the coarse texture of *C. hirtus*, confocal (405 nm and 561 nm), two-photon and aptome techniques all have extremely high signal-to-noise ratios (0.99; see Figure 5) but that widefield fluorescence has a much lower signal-to-noise ratio (0.76; see Figure 5). However, following deconvolution, the recovery of textural information from *C. hirtus* using the widefield technique is substantially improved. This is highlighted by the detailed images of *C. hirtus* in Figure 5, and also by differences in the smoothness of the intensity profiles of each technique. The intensity profile of the widefield technique coupled with deconvolution is much smoother than the intensity profiles of raw data from confocal, two-photon and apotome microscopy, and this is particularly clear at the apex of each peak in the intensity profiles (Figure 5).

The impact of deconvolution is also visible in the line profiles of pixel intensity values of the NDL texture of *A. repens*. Intensity profiles from 405 nm and 561 nm confocal and two-photon microscopy all show substantial increases in the amplitude of each peak and trough following deconvolution (Figure 5). The granulate texture of *A. repens* is visible using 405 nm confocal microscopy (Figure 3 and Video S2), and the deconvolution of the widefield microscopy image improved the texture of this pollen (Figure 5).

## Discussion

### Comparison of microscopy techniques

None of the microscopy techniques investigated here provides a complete reconstruction of both the shape and surface texture of all three pollen types. Reflected light techniques that use longer excitation wavelengths (e.g. 780 nm) and multi-photon excitation provide good depth penetration and hence good reconstructions of the shape of pollen grains (Figures 2 & 3) [31], but such techniques fail to resolve NDL surface texture (see Figure 3 two-photon *A. repens*). Reflected light techniques that use shorter wavelengths (e.g. 405 nm) or incoherent light, both only when coupled with deconvolution (e.g. widefield fluorescence) provide reasonable information on NDL texture (Figure 3). However, these techniques either have poor depth penetration (405 nm, in the case of *C. hirtus* and *M. occidentalis*) or suffer considerable convolution (widefield, all pollen species) and hence provide poor reconstructions of the shape of pollen grains (Figures 2 and 3). The relative ability of each microscopy technique to provide information on the shape and surface texture of *C. hirtus*, *M. occidentalis* and *A. repens* is compared in Table 2.

It is notable that widefield fluorescence coupled with deconvolution may provide a picture of coarse surface textures that is comparable to raw data from confocal, two-photon and apotome microscopy (Figure 5). Widefield fluorescence coupled with deconvolution provides a clearer picture of the surface texture of *A. repens* than confocal and two-photon microscopy (Figures 3 & 5). This is unexpected in the case of confocal microscopy because the resolution of this technique is greater than widefield microscopy (confocal FWHM: 360 nm; widefield FWHM: 470 nm) (Figure 1). However, this increase in resolution is offset by the rejection of photons from out of focus light by the confocal pinhole, which discards useful information required for successful deconvolution [30], [32]. Further, the pixel integration time of the confocal system used in this study is considerably smaller than the pixel integration time of the widefield system used in this study (confocal: 1.58 µs; widefield: 219 ms). This gives the widefield system a superior signal-to-noise ratio. The rejection of photons during image acquisition may also explain why the texture of *A. repens* is poorly represented by apotome microscopy (Figure 3) despite the high resolution of this technique (FWHM: 340 nm) (Figure 1), higher pixel integration time, and noted optical sectioning strength of structured illumination widefield fluorescence microscopy [28]. The non-linear gain in resolution of two-photon microscopy, together with the inherent confocality of this technique in the absence of a pinhole, is cancelled by the use of a longer wavelength of light [30], [32], and this explains the failure of this technique to provide information on the NDL texture of *A. repens* (e.g. Figures 3 & 4).

Transmitted light techniques with coherent short wavelength (405 nm laser) illumination seem to provide higher contrast compared to incoherent light illumination (halogen in brightfield) (e.g. Figure 3). Additionally, the exine color of *A. repens* was the lightest of the three pollen types investigated here, and also had the lowest light absorption, whereas exines of *M. occidentalis* and *C. hirtus* were considerably darker in color and had higher absorption (Figures 3 and 7). This may explain why the contrast of *M. occidentalis* and *C. hirtus* in brightfield microscopy is greater than *A. repens*. Differences in contrast transfer functions between coherent and incoherent light and other optical contrast techniques and transfer functions are shown in [33]. Finally, DIC produces images with enhanced contrast when objects have high spatial frequency (see the optical transfer functions shown in Figure S2) [34]. This explains why the NDL texture of *A. repens* is more clearly represented by DIC compared to brightfield microscopy (e.g. Figure 3).

### Absorption of light by the pollen exine affects SNR

**Figure 6:**
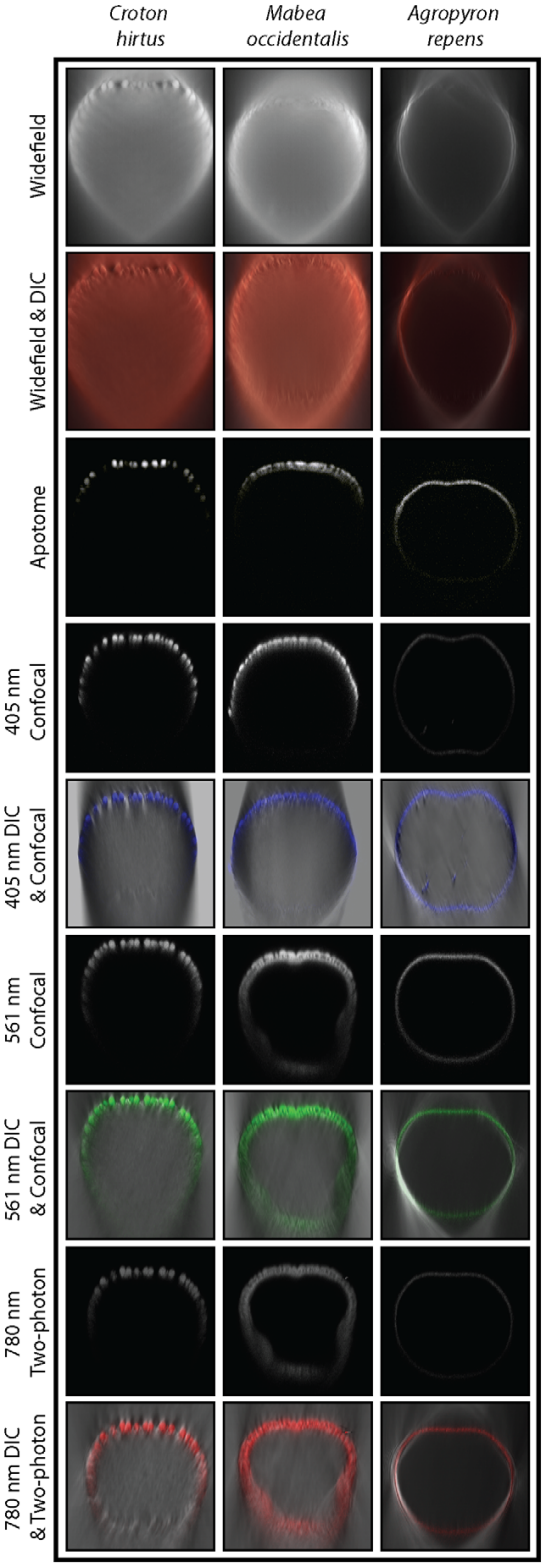
Orthogonal projections to highlight the depth penetration of each optical microscopy technique investigated here. All images show single optical plane approximately from the centre of the Z-stack. In merged reflected and DIC planes, data from reflected light techniques has been pseudocolored according to the excitation wavelength. 405 nm = blue; 561 nm = green; 780 nm = red. The widefield images are red psuedocolored to reflect the rhodamine (546/10) excitation filter. Images from all reflected light techniques were deconvolved except apotome microscopy, which is represented by raw data. Images are displayed in min/max intensity profile. Pollen sizes as for Figure 2. Projections were constructed using the Autoquant slice viewer rendering algorithm.

All the optical microscopy techniques investigated here recover less morphological information from deep planes than surface planes of *C. hirtus*, *M. occidentalis* and *A. repens* (e.g. Figure 3). This is highlighted by a series of orthogonal projection of the three pollen types, which show that as the excitation wavelength decreases, so too does the amount of morphological information recovered from deep within a specimen (Figure 6). This is shown most clearly by *M. occidentalis*: confocal microscopy using 405 nm excitation light provides little information from deep within the specimen, but that the use of 561 nm results in enhanced information recovery from deep planes (Figure 6 and Videos S3, S4).

**Figure 7:**
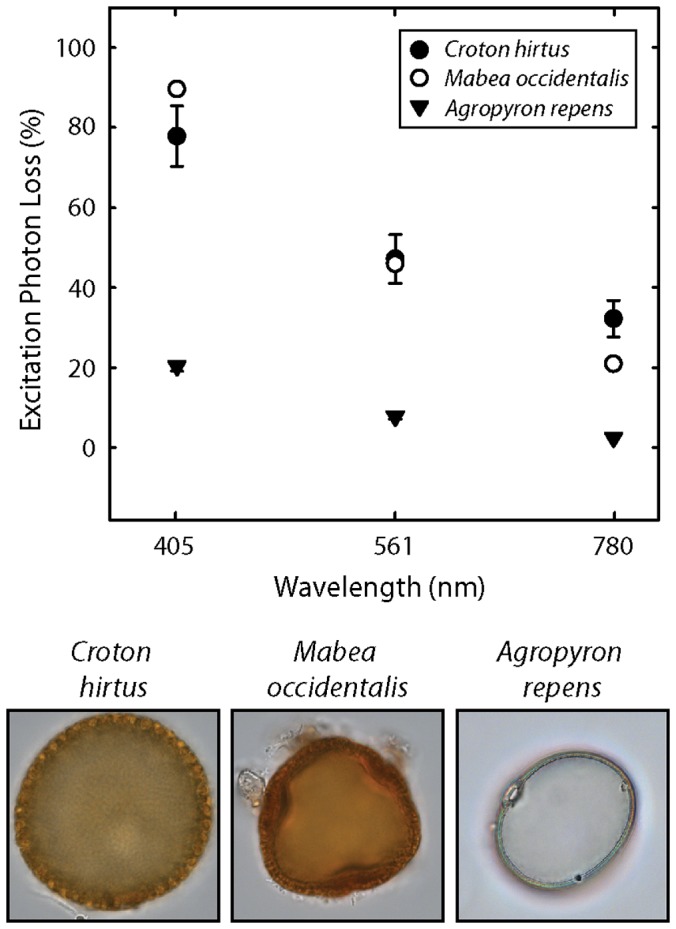
Absorption of light by the pollen exine. Showing measurements of the percentage of light absorbed by the exine of the three pollen types investigated here, together with brightfield images (focused to the middle plane) of these pollen grains. Absorption was calculated by measuring the difference between the intensity of light passing through the mounting media and the intensity of light passing through a pollen grain. Three absorption measurements were made from three specimens per pollen type. Error bars represent maximum and minimum values. Where no error bars are visible, the range of data was less than the size of the symbol.

We interpret this pattern as an expression of the absorption of light by the pollen exine, and in order to investigate this in more detail and to consider its implications for the study of pollen shape, we have measured the absorption of different wavelengths of light by each pollen type. *C. hirtus* absorbs ∼75% of 405 nm light, ∼50% of 561 nm light and ∼30% of 780 nm light (Figure 7). *M. occidentalis* absorbs ∼90% of 405 nm light, ∼50% of 561 nm light and ∼20% of 780 nm light (Figure 7). *A. repens* absorbs ∼20% of 405 nm light, ∼5% of 561 nm light and ∼2% of 780 nm light (Figure 7).

All pollen types absorb a greater proportion of shorter wavelength light than longer wavelength light, which may be related to the presence of compounds in sporopollenin that absorb short-wavelength light such as ultraviolet-B, e.g. [35]. *C. hirtus* and *M. occidentalis* absorb considerably more light of all wavelengths than *A. repens* (Figure 7). This is perhaps because these two pollen types have significantly thicker exines than *A. repens*, as shown by the color brightfield images in Figure 7. Additionally, there is greater variability in the amount of light absorbed by *C. hirtus* than either *M. occidentalis* or *A. repens* (Figure 7). This may be due to the scattering of light by the lens like individual prominent sculptural elements of this pollen type (see Figure 3). These analyses highlight that the recovery of morphological information from pollen grains is dependent not only on the nature and wavelength of the light used to image a pollen grain (e.g. Figures 2,3,4), but also on the nature of the exine of different pollen types. These results suggest that the shape and deep-plane texture of pollen types with thin exines may be recovered with short excitation wavelengths, but those pollen grains with thick exines and very coarse surface sculpture may require longer excitation wavelengths.

### Resolving the unresolved: SR-SIM

**Figure 8:**
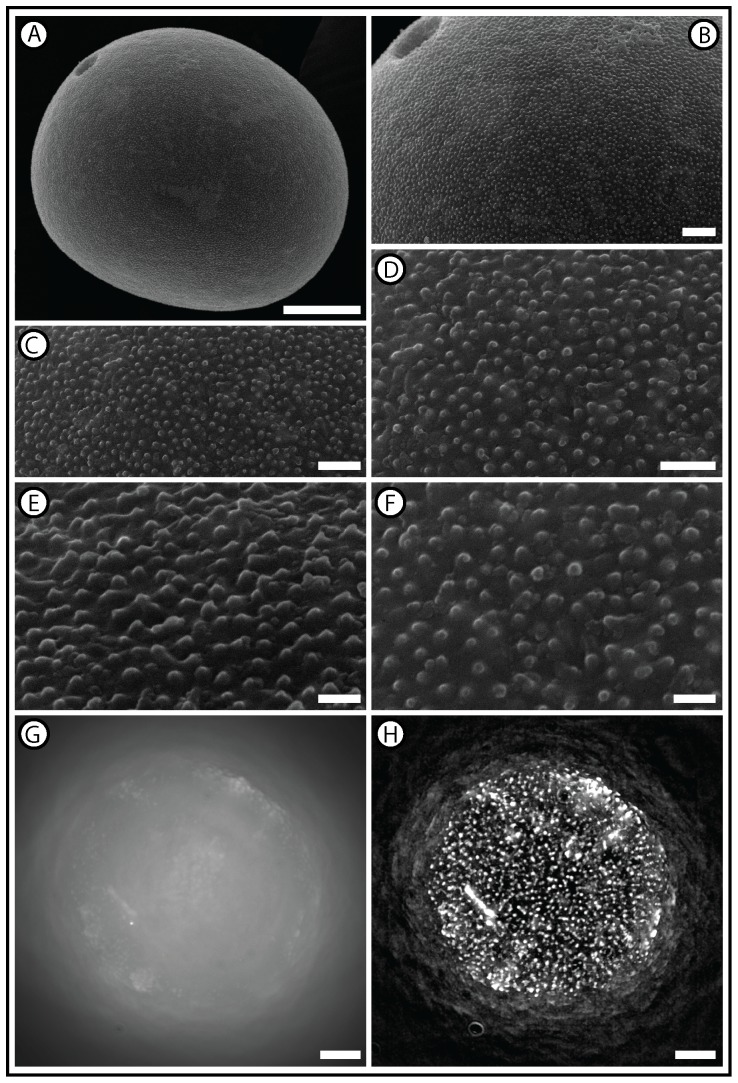
Scanning electron microscopy and super-resolution-SIM images of *Agropyron repens*. Note the surface texture of the pollen grain, which comprises a dense covering of elements that measure ∼250 nm in all directions (granules; [16]). *A*: showing the characteristic sub-circular profile and single pore of *A. repens*; ×2K magnification; scale bar represents 10 µm. *B*: showing details of the surface texture and pore that lacks a prominent annulus; ×6K magnification; scale bar represents 2 µm. *C–F*: showing details of the surface texture of *A. repens* at ×12K (*C*) (scale bar represents 1 µm), ×20K (*D*) (scale bar represents 1 µm) and ×30K (*E* & *F*) (scale bars represent 500 nm). *E* shows an oblique view of the surface texture of *A. repens* and *F* shows a vertical view of the surface texture of *A. repens*. *G* and *H* show widefield and SR-SIM images from the Zeiss Elyra super resolution system. Note that the granules (∼250 nm in all directions) on the surface of *A. repens* are well resolved in *H*.

The diffraction limit of light [36] places a fundamental constraint on the recovery of morphological information from pollen grains. In such situations, the recovery of morphological information using light microscopy relies on the correct interpretation of the emergent pattern of two or more unresolved sculptural elements, or SEM may be employed [23], [37]. Our example of a pollen grain with texture close to the diffraction limit of light is *A. repens*, and a key difference between the images of *A. repens* from SEM and the optical microscopy techniques investigated here is the representation of the surface texture. The high resolution SEM images of *A. repens* show that there are almost no areas of the pollen grain surface characterized by a fine rugulate sculpture (Figure 8), and this indicates that the fine rugulate texture suggested by brightfield, 561 nm DIC and 780 nm DIC (Figures 3 & 4) is likely to be an artifact of the low resolution of these techniques.

In contrast, the surface texture of *A. repens* from the SR-SIM technique appears very similar to the surface texture of *A. repens* from the SEM (Figure 8). There are almost no finely rugulate areas on the surface of the pollen grain, and where granules appear to be connected, the individual granules are clearly defined by sub-circular clusters of high-intensity pixels (Figure 8). This provides an early example of how super-resolution light microscopy techniques might be applicable to problems in palynology where the recovery of very fine textural information is required. The SR-SIM technique may also hold at least two advantages over the SEM: (1) SEM provides information only from the surface of a specimen, whereas SR-SIM provides some shape data; (2) debris in a palynological preparation such as plant cuticle can completely obscure pollen grains on an SEM stub [23], but the SR-SIM may be able to provide some data from such obscured pollen grains, in common with other optical microscopy techniques.

### Concluding Remarks

There appears to be no single optical microscopy technique that can satisfactorily capture both pollen shape and surface texture, and a combination of reflected and transmitted light techniques is required to maximize the recovery of morphological information from pollen grains. An example of such a combination might be the simultaneous acquisition of shape and surface texture data using 561 nm confocal and DIC microscopy. The application of appropriate combinations of techniques should lead to more robust morphological classifications, and an increase in the taxonomic precision with which ancient vegetation can be reconstructed using pollen grains [13], [14]. Based on our analyses, we provide a simplified guide to the imaging of pollen grains using optical microscopy in Table 3. The following specific conclusions are drawn:

1. Reconstructions of the shape of pollen grains are limited by the axial resolution and the depth penetration of the microscopy technique employed. Widefield fluorescence provides reconstructions of pollen shape that taper into a pronounced point (Figure 2) owing to the poor axial resolution of this technique (∼1000 nm) [30], [32]). Confocal microscopy using 561 nm excitation light provides more information on the shape of all three pollen types investigated here than confocal microscopy using 405 nm excitation light (Figures 2, 3 & 6). This is apparently because short wavelengths of light do not penetrate as deeply into pollen grains as long wavelengths of light (Figures 3 & 6), which may be related to the absorption of short wavelength light by the pollen exine (Figure 7). Two-photon microscopy provides appreciable information on the shape of pollen grains (Figures 2, 3 & 6), which is probably due to a combination of long-wavelength excitation light (780 nm) and multi-photon excitation phenomena [31].
2. SEM reveals that the surface texture of *A. repens* comprises a dense sculpturing of granules that measure ∼250 nm in all directions, close to the diffraction limit of light [20], [36] (Figure 8). Among reflected light techniques, widefield fluorescence coupled with deconvolution provides the clearest view of the surface texture of *A. repens* (Figure 3). This is likely because the widefield technique does not reject any photons during image acquisition [30], [32], and because of the high pixel integration time (219 ms), which led to more light-capture in the widefield system used in this study.
3. Among transmitted light techniques, widefield DIC and 405 nm DIC provide the clearest view of the surface texture of *A. repens* (Figures 3 & 4). Under brightfield microscopy, 561 nm DIC and 780 nm DIC the texture of *A. repens* consists of more linear features (e.g. Figure 4). This highlights that the nature and wavelength of incident light have a profound effect on the recovery of fine morphological details from pollen grains, particularly those near the diffraction limit of light.
4. SR-SIM microscopy provides data on the surface texture of *A. repens* that is more comparable to textural data from the SEM than any other light microscopy technique investigated here (Figure 8, Figure S3 and Video S7). This provides an example of a super-resolution light microscopy technique that is able to accurately resolve morphological details of pollen grains that stretch the capabilities of conventional optical microscopy techniques.

**Table 3:** Simplified guide to the imaging of pollen grains using optical microscopy.

	Shape	Morphological Details Furthest From Cover-Glass	Surface Texture	
			*Coarse*	*Fine (NDL)*
**Reflected Light**	Long wavelength and/or multi-photon excitation (e.g. two-photon 780 nm)	Long wavelength and/or multi-photon excitation (e.g. two-photon 780 nm)	Intermediate wavelength, multi-photon excitation not required (e.g. confocal 561 nm)	Short wavelength (e.g. confocal 405 nm) or SR-SIM
**Transmitted Light**	Not Recommended	Long/intermediate wavelength and contrast enhancement (e.g. DIC 780/561 nm). Short wavelengths (e.g. 405 nm) may not penetrate through the entire pollen exine	Intermediate wavelength or incoherent light and contrast enhancement (e.g. DIC 561 nm or widefield DIC with halogen illumination)	Short wavelength and contrast enhancement (e.g. DIC 405 nm)

As a rule of thumb, an increase in the wavelength of incident light results in a decrease in resolution. Consequently, shorter wavelengths of light are more suitable for imaging fine surface textures, including those near the diffraction limit of light (NDL; ∼250 nm). Super-resolution structured illumination microscopy (SR-SIM) provides textural information comparable to the SEM. Longer wavelengths of light penetrate further through the pollen exine than shorter wavelengths, and hence are more suitable for reconstructing the shape of pollen grains and capturing morphological information from optical sections furthest from the cover-glass.

## Materials and Methods

### Ethics Statement

No permits were required for the described experiments. Pollen samples were taken from herbarium specimens, so there was no collection of living material. The *Agropyron repens* grains used in this analysis were from the pollen residues from the University of Maryland; these residues had been isolated from accessioned herbarium specimens from the University of Minnesota and the University of Illinois Herbarium. The *Mabea occidentalis* and *Croton hirtus* grains used were isolated with permission from accessioned herbarium samples at the Smithsonian Tropical Research Institute, Panama. None of the three species is listed as endangered or threatened by the IUCN.

### Description of pollen grains

*C. hirtus*, *M. occidentalis* and *A. repens* are morphologically very different to one another. In the following descriptions the terminology follows [16]. *C. hirtus* is ∼50 µm in diameter, circular in outline and inaperturate. The texture of *C. hirtus* is a distinctive Croton pattern, which comprises rings of five or six (occasionally more) raised triangular sculptural elements arranged around a circular area. *M. occidentalis* is ∼50 µm in diameter, sub-circular in outline and tricolpate. The texture of *M. occidentalis* is foveolate, and comprises numerous rounded depressions or lumina ∼1 µm in diameter. *A. repens* is ∼40 µm in diameter, sub-oval in outline and has a single pore surrounded by a weakly developed annulus. The texture of *A. repens* is granulate, and comprises a dense covering of raised elements that measure ∼250 nm in all directions (near the diffraction limit of light; NDL).

### Preparation of pollen grains for light microscopy, SEM and SR-SIM

Pollen of *C. hirtus* and *M. occidentalis* was collected from the herbarium at the Smithsonian Tropical Research Institute, Panama and prepared by standard acetolysis following [22]. Pollen of *A. repens* was collected and prepared by [38]; treatment comprised immersion with HCl followed by KOH and HF [38]. Following preparation, pollen grains of *C. hirtus*, *M. occidentalis* and *A. repens* were dehydrated in tertiary butyl alcohol and transferred to silicone oil. Two slides of each pollen type were made. Pollen grains were mounted in 10 µl of silicone oil (refractive index 1.526) and covered with a Zeiss high performance number 1.5 cover glass (18×18 mm, thickness 0.170±0.005 mm).

Specimens of *A. repens* were prepared for SEM by mounting individual undehydrated pollen grains from ddH_2_O onto a double-sided adhesive carbon disk that was attached to an SEM stub. The stub was then coated with gold-palladium using a sputter coater, and specimens were viewed using a JEOL JSM-6060LV scanning electron microscope at 25 kV.

Specimens of *A. repens* were prepared for SR-SIM by mixing individual undehydrated pollen grains from ddH_2_O into Prolong Gold (Life Technologies, Carlsbad, CA) anti-fade mounting media. This solution was pipettted onto a Zeiss high performance number 1.5 cover glass (18×18 mm, thickness 0.170±0.005 mm) to allow the ddH_2_O to evaporate. The solution and cover glass were then mounted on a slide and left at room temperature for over 24 hours to allow the mounting media to cure; this raises the refractive index of the mounting media to >1.45 (Life Technologies, Carlsbad, CA). The images were obtained by using 405 nm and 488 nm excitation wavelengths with a Zeiss 100× α-Planapochromat 1.46NA objective at a pixel resolution of 30 nm.

### Widefield fluorescence, apotome and DIC microscopy

Details of the systems, objectives, voxel dimensions, excitation and emission wavelengths, detectors, deconvolution methods, image analysis and rendering are found in Table 1. Details of the microscopy techniques are elaborated below. The apotome optical sectioning system was coupled with X-Cite 120 illumination (Lumen technologies, Ontario, CA). Brightfield and DIC transmitted light images were acquired after setting Köhler illumination using standard DIC optics, and for DIC image acquisition the Nomarski prism bias was kept at the same position to minimize variations. In the case of phase contrast microscopy, an LD Plan-Neofluoar 40×/0.6NA Korr Ph 2 was used after setting the phase annulus to Köhler illumination [39]. Voxel dimensions of the images were kept at 0.1×0.1 µm in the XY direction and 0.3 µm in the Z direction fulfilling the Nyquist sampling criteria. Widefield, apotome and DIC images were cropped at 780×780 pixels, keeping the pollen in the center, to perform deconvolution in one block.

### Single photon, two-photon fluorescence and DIC confocal laser scanning microscopy

Details of the systems, objectives, voxel dimensions, excitation and emission wavelengths, detectors, deconvolution methods, image analysis and rendering are found in Table 1. Details of the microscopy techniques are elaborated below. Beams of the 405 nm (invisible) and 561 nm (visible) single photon diode lasers were split using notch filters, and the pre-chirped 780 nm two-photon laser (MaiTai, DeepSee eHP, Spectraphysics, CA) was split using a 760 nm short pass filter. Since lasers are inherently polarized, an analyzer was not used during the collection of DIC images. Instead, only a pair of Normarski prisms and a polarizer was used. DIC images were acquired simultaneously with the reflected light images using a TPMT module (Zeiss, Obercohen, Germany) after setting the Köhler illumination with a fully opened condenser aperture (0.55 NA). Sampling was performed at 0.1×0.1 µm steps in the XY direction and 0.3 µm steps in the Z direction to fulfill the Nyquist sampling criteria [40], [41]. Multiple two-dimensional image stacks 512×512 pixels were taken along the Z-axis (referred to as Z-stacks). Auto Z correction (linear extrapolation of laser power and master gain) was used for all pollen grains to correct the loss of signal while scanning through the pollen grain. Whenever possible, the same pollen was used to compare different techniques. Photo-bleaching was insignificant as the pollen types investigated here were strongly autofluorescent. In some experiments, the images were scanned sequentially in frame mode using all three wavelengths (405, 561 and two photon 780 nm) simultaneously on the same pollen.

### Point spread functions (PSF)

Point spread functions were measured on all optical reflected microscopic techniques to assess their individual performance using an orange-red 0.17 mm fluorescent PS speck beads (0.175±0.005 µm, Life Technologies, Carlsbad, CA) under similar excitation and emission parameters described in Table 1 used for pollen samples. The beads were mounted in Prolong Gold anti-fade mounting media. Theoretical/Estimated PSFs were generated while performing deconvolution for each technique. Line profiles were made to verify the shape of the PSF using Autoquant (Mediacybernatics, Bethesda, MD) or Axiovision software (Carl Zeiss, Obercohen, Germany).

### Absorption, SNR, FWHM and line intensity profile measurements

Excitation photon loss was measured by placing a light sensor S121b (400–1100 nm) coupled with a power meter PM 100 (Thorlabs, Gmbh, Dachau, Germany) on a custom designed sensor holder, which could be moved on the specific pollen of interest on the slide. The sensor was lowered to the surface of the slide and the measurements were made while focusing to the middle of the pollen on the other side (inverted scope) using a 10× Planapochromat 0.4NA objective (Carl Zeiss, Obercohen, Germany). Absorption measurements were made from three specimens of each pollen type and expressed as a percentage of background intensity. The signal to noise ratio was measured as described below, where the mean background intensity from raw image data was measured against the mean signal intensities from six different randomly selected regions of a pollen grain. The ratios were averaged for six different positions per sample. Line profiles were created in the program Axiovision (Carl Zeiss, Obercohen, Germany) measurement module. Single optical section (fluorescence) or focal plane (DIC) images were used to generate line profiles. For better comparison, the images were cropped to comparable pixel dimension of around 512×512 pixels. A straight line (default width 1 pixel in the program) was drawn through either center of the image, a single Croton structure or a randomly selected area with high contrast. For images taken from the same pollen grain, the individual planes were assembled as one image stack and the line profiles drawn exactly over the two frames. Pollen grains occasionally moved slightly while switching between two techniques due to the low viscosity of the mounting media. In such cases, line profiles were picked from similar areas but not with identical XY pixel registration.

Orthogonal projection images of pollen were made using the Autoquant -rendering algorithm to visualize the depth penetration in reflected light and how the absorption affected the transmission in DIC images. Single plane images were zoomed in also to assess the resolution. At least three pollen grains were used for each technique and the best representative pollen was shown and further analyzed. The experiments were performed on a single pollen type on the same day to minimize the lamp/laser age, image acquisition room temperature and light path hardware alignment differences.

### Deconvolution

In two-dimensional optical sections, voxel convolution was removed and out-of-focus light was restored using a commercially available blind deconvolution algorithm (Autoquant version ×2.2, Mediacybernatics, Bethesda, MD) originally developed by [40], [41]. This algorithm multiplies the image of perfect fidelity with a PSF calculated from the supplied data (Figure S4), imaging parameters and noise level in the image. Raw Z-stack data from widefield, confocal and two-photon reflected light microscopy was deconvolved using a blind deconvolution algorithm. Each technique was matched to the correct modality (wide-field, laser scanning confocal and multiphoton modalities), and 10 constrained iterations with low/medium noise setting were performed. Deconvolution was not performed on data from apotome microscopy.

### Display of Optical Sections

From each pollen type, a single optical surface plane (clearest view of the texture closest to the cover glass) and deep plane (closest match to the surface plane texture but furthest from the cover glass) was selected after deconvolution. The final images were presented under min/max or best-fit (SIM data only) intensity profile. Minimum and maximum pixel intensities were displayed on a scale of 0–256 grey levels (8-bit). The X-Y, X-Z (maximum intensity projection; e.g. Figure 2) and orthogonal projection of raw and deconvolved data (median plane; e.g. Figure 6) was accomplished by the Autoquant maximum-intensity projection or slice viewer algorithms. Movies of the DIC images showing individual planes were made in Zeiss Zen 2010 program. Movies of reflected light maximum intensity projections and slice views were rendered using the Surpass Volume module in the Imaris suite software (Bitplane, Zürich, Switzerland). Figures were preassembled in Adobe Photoshop and Illustrator (Adobe systems, San Jose, CA).

## Supporting Information

Figure S1 **Performance of phase contrast technique in revealing the pollen morphology.** The phase contrast technique failed to provide textural details of the pollen grains for all three species, shown from top to bottom, *A. repens*, *C. hirtus* and *M. occidentalis*.
(TIF)

Figure S2 **Optical transfer function differences between brightfield and DIC.** The NDL surface texture of *A. repens* is better revealed under DIC technique compared to brightfield. DIC produces images with enhanced contrast when objects have high spatial frequency and this could be explained by the differences in the optical transfer function. Retraced from [34].
(TIF)

Figure S3 **Super-resolution structured illumination microscopy (SR-SIM) could resolve NDL surface texture easily.** Screenshot of a line profile over three surface textures of super resolution-SIM data showing the FWHM of around 244 nm. No other reflected light technique tested provided such high signal to noise ratio and resolution.
(TIF)

Figure S4 **Theoretical point spread functions used in blind deconvolution algorithms.** Estimated PSFs of wide-field, confocal laser scanning and multiphoton modalities used for deconvolving the pollens using the blind deconvolution in the program Autoquant. The intensity profiles were measured to determine the psf shape at the single plane in the center of the stack. Note the wide field yielded the broader FWHM, than the confocals as expected and the confocal and multiphoton modalities yielded similar PSF. Gamma values over 8.0 was used to visualize the intensity distribution and PSF shape.
(TIF)

Video S1 **3D rendering of** ***A. repens*** **pollen imaged using wide-field microscopy.** The first part is a series of optical sections, and the second part is a 3D projection of all slices after blind deconvolution.
(AVI)

Video S2 **3D rendering of** ***A. repens*** **pollen having NDL structures imaged using 405 nm laser scanning confocal microscopy.** The first part is a series of optical sections, and the second part is a 3D projection of all slices after blind deconvolution.
(AVI)

Video S3 **3D rendering of** ***M. occidentalis*** **pollen imaged using 405 nm laser scanning confocal microscopy.** The first part is a series of optical sections, and the second part is a 3D projection of all slices after blind deconvolution.
(AVI)

Video S4 **3D rendering of** ***M. occidentalis*** **pollen imaged using 561 nm laser scanning confocal microscopy.** The first part is a series of optical sections, and the second part is a 3D projection of all slices after blind deconvolution.
(AVI)

Video S5 **3D rendering of** ***C. hirtus*** **pollen showing large lens like features imaged using aptome optical microscopy technique.** The first part is a series of optical sections, and the second part is a 3D projection of all slices.
(AVI)

Video S6 **3D rendering of** ***C. hirtus*** **pollen showing large lens like features imaged using two-photon confocal microscopy using the 780 nm excitation.** The first part is a series of optical sections and the second part is a 3D projection of all slices after blind deconvolution.
(AVI)

Video S7 **3D rendering of** ***A. repens*** **pollen images using super-resolution structured illumination microscopy (SR-SIM).** All images are stacked and the movie is a 3D projection of all optical sections.
(WMV)

Video S8 **2D image stacks of** ***A. repens*** **under widefield transmitted light DIC technique.** Images are rendered in Zeiss Zen software and the depth of each plane is shown in top left corner.
(AVI)

Video S9 **2D image stacks of** ***C. hirtus*** **under widefield transmitted light DIC technique.** Images are rendered in Zeiss Zen software and the depth of each plane is shown in top left corner.
(AVI)

Video S10 **2D image stacks of** ***A. repens*** **under confocal 405 nm excitation transmitted light DIC technique.** Images are rendered in Zeiss Zen software and the depth of each plane is shown in top left corner.
(AVI)

Video S11 **2D image stacks of** ***C. hirtus*** **under confocal 405 nm excitation transmitted light DIC technique.** Images are rendered in Zeiss Zen software and the depth of each plane is shown in top left corner. This image was taken with a photomultiplier gain gain that is two-fold greater than the other images.
(AVI)

Video S12 **2D image stacks of** ***A. repens*** **under confocal 561 nm excitation transmitted light DIC technique.** Images are rendered in Zeiss Zen software and the depth of each plane is shown in top left corner.
(AVI)

Video S13 **2D image stacks of** ***C. hirtus*** **under confocal 561 nm excitation transmitted light DIC technique.** Images are rendered in Zeiss Zen software and the depth of each plane is shown in top left corner.
(AVI)

Video S14 **2D image stacks of** ***A. repens*** **under two photon 780 nm excitation transmitted light DIC technique.** Images are rendered in Zeiss Zen software and the depth of each plane is shown in top left corner.
(AVI)

Video S15 **2D image stacks of** ***C. hirtus*** **under two photon 780 nm excitation transmitted light DIC technique.** Images are rendered in Zeiss Zen software and the depth of each plane is shown in top left corner.
(AVI)
