# Supplementary figures and images for: Capturing the Surface Texture and Shape of Pollen: A Comparison of Microscopy Techniques
Source: PLoS One. 2012 Jun 12;7(6):e39129. doi: 10.1371/journal.pone.0039129 (PMC3373610; doi:10.1371/journal.pone.0039129)

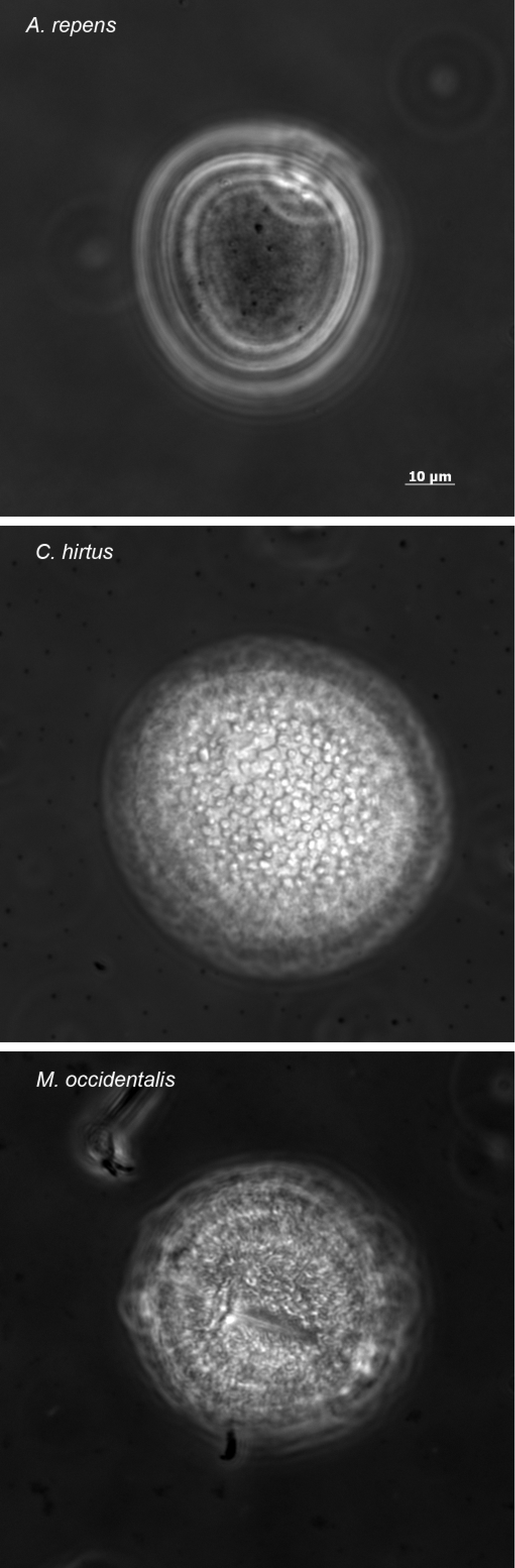

Supplement: Figure S1 — Performance of phase contrast technique in revealing the pollen morphology. The phase contrast technique failed to provide textural details of the pollen grains for all three species, shown from top to bottom, A. repens, C. hirtus and M. occidentalis. (TIF) [file pone.0039129.s001.tif]

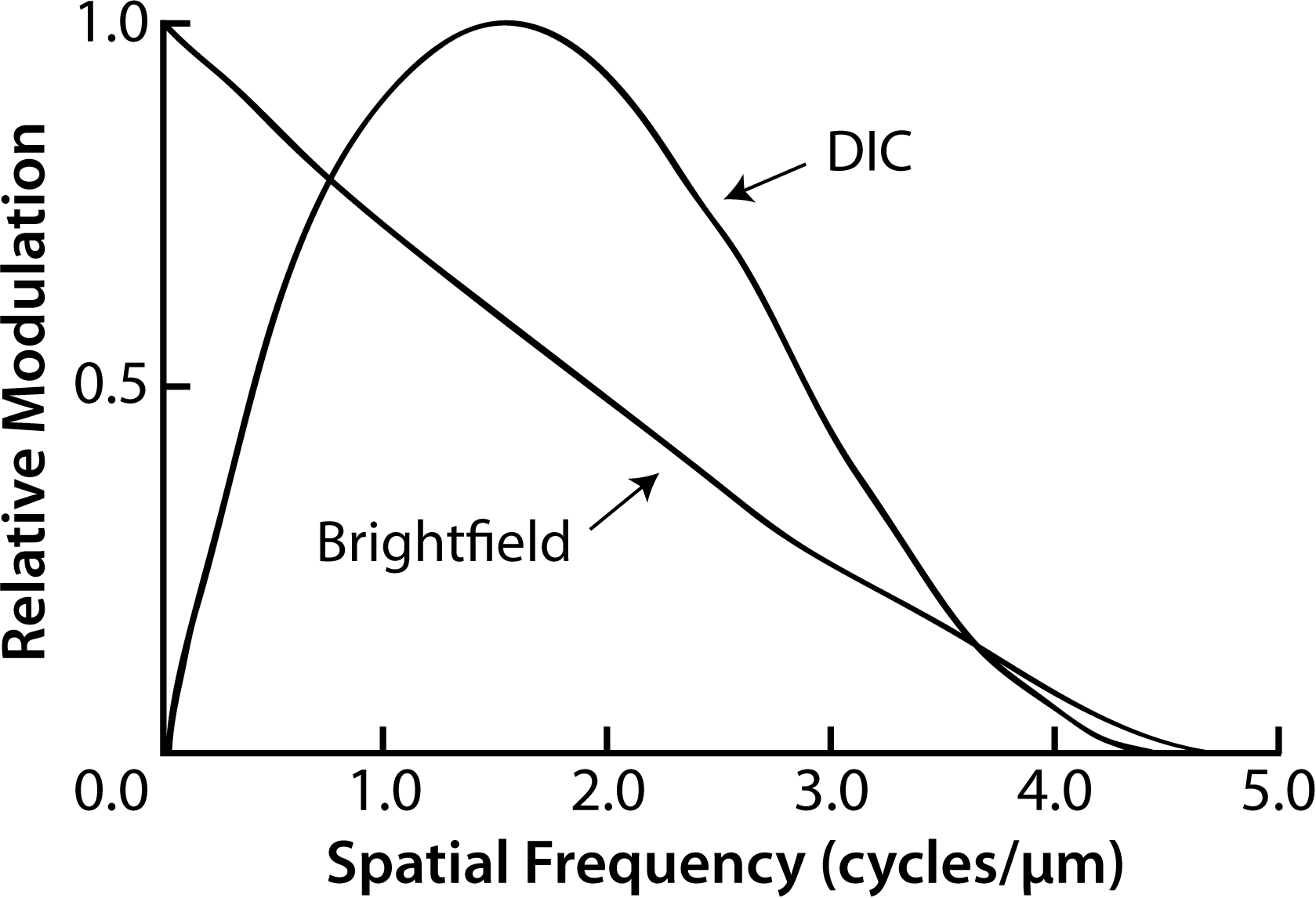

Supplement: Figure S2 — Optical transfer function differences between brightfield and DIC. The NDL surface texture of A. repens is better revealed under DIC technique compared to brightfield. DIC produces images with enhanced contrast when objects have high spatial frequency and this could be explained by the differences in the optical transfer function. Retraced from [34]. (TIF) [file pone.0039129.s002.tif]

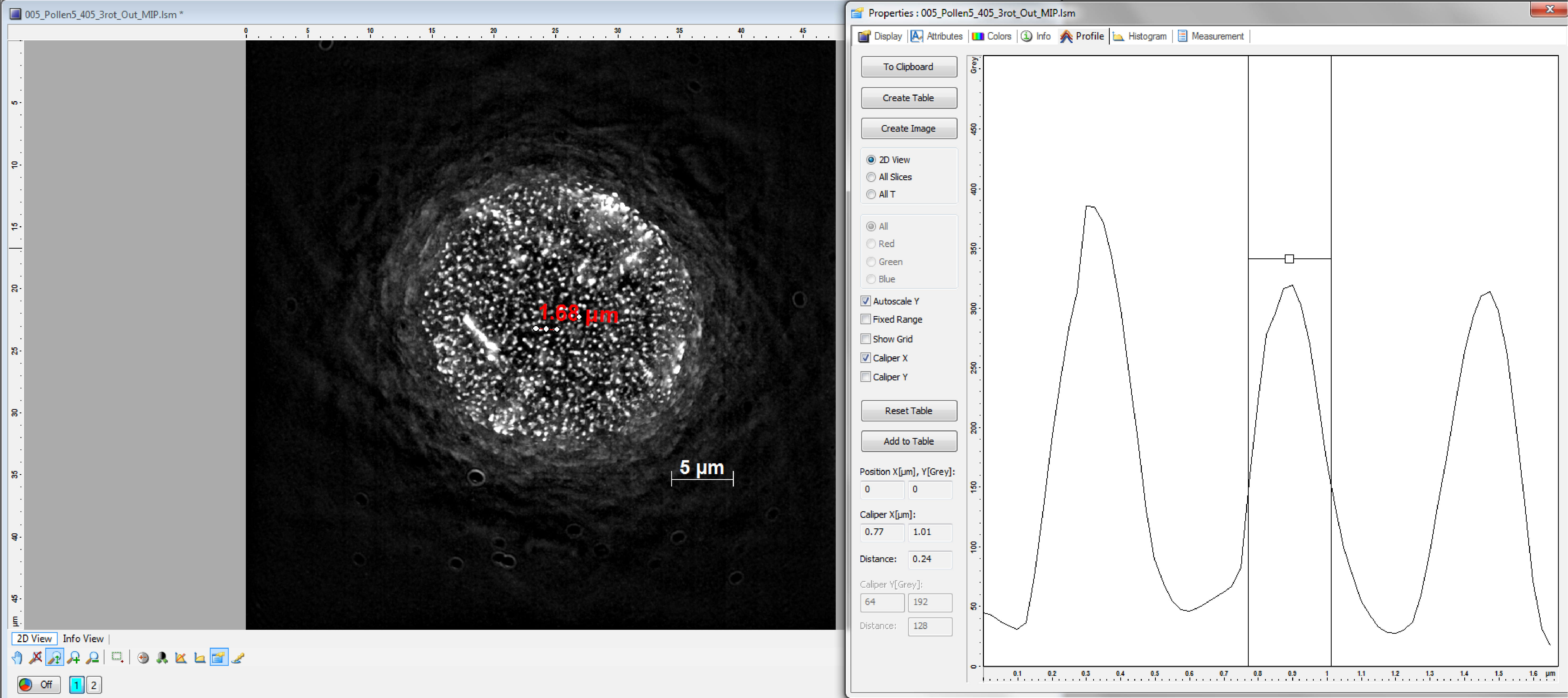

Supplement: Figure S3 — Super-resolution structured illumination microscopy (SR-SIM) could resolve NDL surface texture easily. Screenshot of a line profile over three surface textures of super resolution-SIM data showing the FWHM of around 244 nm. No other reflected light technique tested provided such high signal to noise ratio and resolution. (TIF) [file pone.0039129.s003.tif]

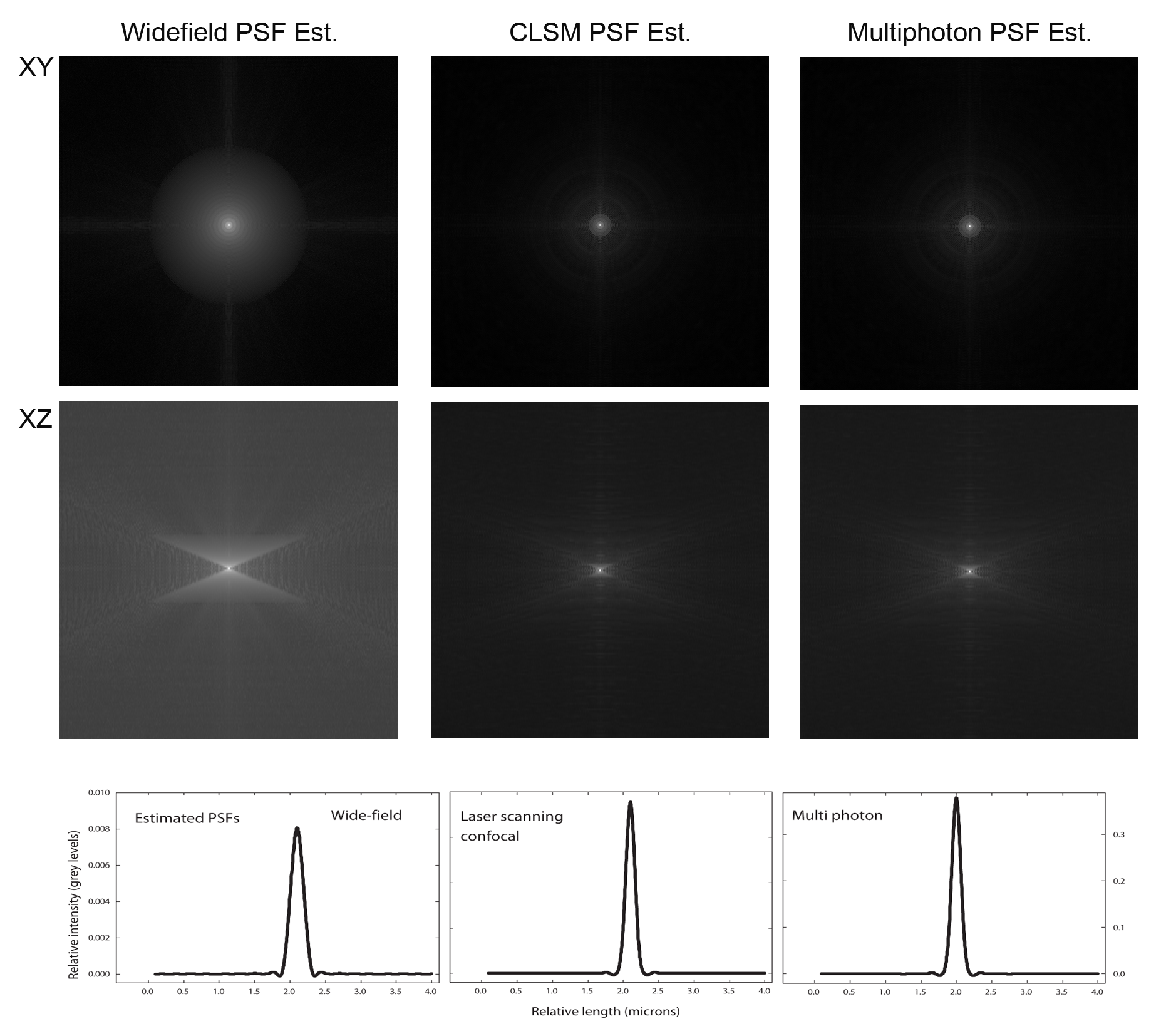

Supplement: Figure S4 — Theoretical point spread functions used in blind deconvolution algorithms. Estimated PSFs of wide-field, confocal laser scanning and multiphoton modalities used for deconvolving the pollens using the blind deconvolution in the program Autoquant. The intensity profiles were measured to determine the psf shape at the single plane in the center of the stack. Note the wide field yielded the broader FWHM, than the confocals as expected and the confocal and multiphoton modalities yielded similar PSF. Gamma values over 8.0 was used to visualize the intensity distribution and PSF shape. (TIF) [file pone.0039129.s004.tif]
